# Mitochondrial dysfunction in myasthenia gravis: Exploring directions for future immunotherapy? A review

**DOI:** 10.17305/bb.2024.11197

**Published:** 2024-10-01

**Authors:** Jianan Chen, Jing Lu, ZhiGuo Lv, Baitong Wang, Shanshan Zhang, Peng Xu, Jian Wang

**Affiliations:** 1 The School to Changchun University of Chinese Medicine, Jilin, Changchun, China; 2 Research Center of Traditional Chinese Medicine, The Affiliated Hospital to Changchun University of Chinese Medicine, Jilin, Changchun, China; 3 Department of Encephalopathy, The Affiliated Hospital to Changchun University of Chinese Medicine, Jilin, Changchun, China

**Keywords:** Myasthenia gravis, mitochondria, immunity, metabolism, pharmacological effects

## Abstract

Myasthenia gravis (MG) is an acquired autoimmune disease characterized by impaired transmission at the neuromuscular junction, primarily manifesting as fluctuating muscle weakness, fatigability, and partial paralysis. Due to its long disease course, treatment resistance, and frequent relapses, it places a significant burden on patients and their families. In recent years, advances in molecular biology have provided growing evidence that mitochondrial dysfunction impairs muscle function and affects immune cell proliferation and differentiation in patients. Mitochondria, as the cell’s energy source, play a critical role in various pathological processes in MG, including oxidative stress, dynamic abnormalities, mitophagy, and mitochondrial metabolism. The role of mitochondrial dysfunction in the pathogenesis of MG has garnered increasing attention. This manuscript primarily explores mitochondrial function and abnormal morphological changes in MG, as well as mitochondrial quality control, metabolic reprogramming, and their potential mechanisms in the pathological changes of the disease. It also reviews the current status of drug therapies aimed at improving mitochondrial function. The goal is to provide novel perspectives and strategies for future mitochondrial-targeted therapies in MG.

## Introduction

Myasthenia gravis (MG) is an autoimmune neurological disorder caused by autoantibodies that impair transmission at the neuromuscular junction (NMJ). The exact mechanisms that trigger the immune response remain unclear. Epidemiological data [1, 2] indicate a global prevalence of 150–250 cases per million, with an annual incidence rate of 1.7–30 per million, showing an increasing trend over the years. The primary clinical symptoms of MG include fluctuating and fatigable muscle weakness, which can affect skeletal muscles throughout the body, particularly those of the eyes, bulbar region, and limbs. Clinical manifestations vary depending on the type of autoantibodies involved and the presence of a thymoma. In severe cases, the condition can be life-threatening. Research indicates that various immune cells contribute to the pathogenesis of MG [3]. The imbalance of CD4^+^ T cell subsets plays a critical role in the development of MG in both patients and experimental autoimmune MG (EAMG) mice. Dendritic cells (DCs) are key in linking innate and adaptive immune responses and are essential for activating T cells that target the body’s tissues [4]. In MG, these activated antigen-specific T cells aid B cells in producing various autoantibodies [5]. In patients with MG, autoantibodies target the postsynaptic membrane at the NMJ, reducing the density of acetylcholine receptors (AChRs) or nonspecifically binding to AChRs on the postsynaptic membrane. This disrupts acetylcholine from binding effectively to the receptors [6, 7], leading to reduced endplate potential and impaired signal transmission at the NMJ.

Recent studies on MG have identified seven additional pathogenic antibodies beyond AChR antibodies, which attack various extracellular or intracellular targets [8]. In some patients, no antibodies are detectable in their serum. Treatment of MG faces significant challenges. Cholinesterase inhibitors are primarily used as first-line symptomatic treatments, while corticosteroids, used as second-line therapies, are often administered alone or combined with first-line drugs. However, these treatments rarely achieve complete and sustained symptom relief. For young patients with AChR-positive MG or thymomas, thymectomy may be considered. Additionally, critically ill patients may undergo plasmapheresis or immunoglobulin therapy [9]. Despite these options, around 10% of MG patients are considered refractory to immunosuppressive therapy [10, 11]. In these cases, adequate doses and prolonged use of corticosteroids and at least two immunosuppressants fail to stabilize the condition. Symptoms either persist or worsen, often accompanied by side effects that limit functionality [12]. Some patients may experience a temporary worsening of symptoms within 2–3 weeks of corticosteroid treatment, potentially triggering a myasthenic crisis, especially in cases of late-onset, severe disease, or prominent bulbar symptoms. Long-term use of corticosteroids carries substantial side effects [13]. Thus, there is an urgent need to explore new treatments that can inhibit the immunopathological changes in MG and halt disease progression.

Mitochondria, the energy factories of cells, are essential for normal cellular operation. Besides generating energy through oxidative phosphorylation (OXPHOS), they regulate various biological processes, including cell signaling, apoptosis, and programmed cell death. Mitochondria maintain a dynamic equilibrium through fusion and fission [14] ensuring their structural integrity and proper function. Dysfunction in mitochondria can have severe consequences [15]. While earlier research on MG focused primarily on immune mechanisms, recent studies have highlighted the significant role mitochondria play in the disease. Mitochondrial structural abnormalities and dysfunction have been observed in the muscles and immune cells of both MG patients and EAMG rats. Mitochondrial issues in MG primarily involve problems with transport and distribution, fusion and fission, biogenesis, autophagy, oxidative stress, and metabolic reprogramming. This paper will explore the impact of mitochondrial dysfunction on MG and its specific pathophysiological mechanisms, as well as potential drugs for mitochondrial-targeted therapy in MG.

### Structural abnormalities of mitochondria in MG

Mitochondria, believed to share a common ancestor with bacteria, have a double-membrane structure, which sets them apart from other organelles. They are typically rod-shaped or spherical and consist of four functional regions: the outer mitochondrial membrane, the intermembrane gap, the inner mitochondrial membrane, and the matrix. The outer membrane is smooth and forms the boundary of the organelle, while the inner membrane folds inward to form cristae, which are crucial for various biochemical reactions. These membranes are arranged in parallel and exhibit typical unit membrane characteristics [16]. The mitochondrion is divided into two compartments by these membranes: the intermembrane space and the matrix, which houses proteins critical for mitochondrial functions [17]. Mitochondria are constantly moving and undergoing fusion and fission, creating a dynamic network. Under normal conditions, mitochondria maintain an intact structure, with prominent cristae, complete membranes, and evenly distributed chromatin, as seen under ultrastructural observation.

In the skeletal muscles of EAMG rats, pathological changes occur, including disrupted myofibrils and mitochondria, ruptured mitochondrial membranes, and blurred and irregular cristae, with some appearing as empty vesicle-like structures. Mitochondrial ultrastructural changes are observed in rodents within 24 h of the passive transfer of AChR antibodies [18] and within seven days after active induction of AChR-EAMG [19]. In passively immunized AChR-EAMG muscle tissues, extraocular muscles (EOMs) exhibit more significant lymphocyte infiltration and more pronounced mitochondrial abnormalities compared to limb muscles and the diaphragm. The weakness of the EOMs is often an early symptom of MG, and they typically respond well to standard immunotherapy. However, some MG patients develop refractory ophthalmoplegia. For example, in sub-Saharan Africa, the incidence of severe, persistent, and refractory ocular paralysis in MG is higher than the global average. In the most severe cases, all EOMs become completely paralyzed, a condition known as ophthalmoplegic MG (OP-MG) [20]. The severity of pathological changes correlates with the degree of muscle paralysis. In cases of EOM paralysis from any cause, there are visible fibrofatty replacement and mitochondrial changes. Developing new methods to treat and prevent MG progression by targeting mitochondrial dysfunction, especially in the EOMs, is essential for future advancements. Genetic analysis of OP-MG patients [21, 22] has revealed that genes associated with mitochondrial ultrastructural integrity and oxidative metabolism are heavily represented in dysregulated gene networks. These findings suggest that there is interference and crosstalk between genes involved in muscle atrophy, regeneration, and mitochondrial oxidative metabolism in the EOMs of MG patients.

Among MG patients, there is a subgroup with muscle-specific kinase (MuSK) antibody positivity. Although these patients represent only about 5% of all MG cases, they often experience acute or subacute onset and rapid disease progression, leading to tongue muscle atrophy and skeletal muscle fasciculations [1, 23]. The observation of muscle atrophy confirms a direct causal link between MuSK antibody-positive MG and muscle cell loss. MuSK-MG causes muscle weakness by disrupting agrin-induced AChR clustering at the NMJ. Besides affecting AChRaggregation on the muscle endplate, anti-MuSK antibodies downregulate key postsynaptic genes and proteins, induce cell cycle arrest, and inhibit cell proliferation, disrupting muscle regeneration and leading to muscle atrophy [24]. Muscle samples from MuSK-MG patients show COX-negative fibers, mitochondrial swelling, degradation, and fragmented cristae, indicating mitochondrial dysfunction. Although AChR-MG does not show significant ultrastructural damage, immunohistochemical analysis reveals greater abnormalities in mitochondrial enzyme activity in AChR-MG [25]. In non-ocular muscles, denervation leads to structural changes in muscle fibers, mainly type II fiber atrophy, regardless of antibody subtype. Type II fibers have fewer mitochondria than type I fibers, which may explain their increased sensitivity to mitochondrial stress. However, this phenomenon is not fully understood and requires further research.

### Functional abnormalities of mitochondria in MG

Both genetic and morphological observations suggest mitochondrial structural abnormalities in MG, indicating associated functional abnormalities. A key aspect of mitochondrial function is maintaining its structural and functional integrity to meet the energy demands of the cell. Mitochondria are highly dynamic organelles, varying in function depending on cell type, and they perform a variety of interconnected roles. These organelles undergo continuous and often reversible physiological recalibrations. Commonly referred to as the “powerhouses” of the cell, mitochondria are essential for numerous physiological processes critical for cellular health and survival, such as redox reactions, calcium homeostasis, ATP synthesis, apoptosis, mitophagy, and the regulation of cellular and environmental stress responses. Changes in mitochondrial function, including impaired quality control and increased oxidative stress, contribute to the muscle weakness and fatigue characteristic of MG. These mitochondrial dysfunctions may also exacerbate the inflammation and immune dysregulation seen in MG, further impairing neuromuscular transmission. Understanding mitochondrial alterations is vital for identifying therapeutic targets, as restoring mitochondrial health may lead to new strategies for managing MG and improving patient outcomes.

#### Mitochondrial quality control and MG

Maintaining mitochondrial function and adapting to fluctuating energy demands are regulated by mitochondrial quality control. This process coordinates mitochondrial biogenesis, dynamics, and autophagy to ensure proper function, including isolating and removing damaged mitochondria [16]. Mitochondrial quality control helps cells manage external stress while producing healthy mitochondria to maintain normal cellular function and prevent disease. Recent evidence has increasingly highlighted the critical role of mitochondrial quality control in MG. Mitochondrial dysfunction not only disrupts energy metabolism but also increases the production of reactive oxygen species (ROS), triggering cellular stress and inflammatory responses that accelerate the pathological progression of MG. Furthermore, the dysregulation of quality control mechanisms like mitophagy can reduce the regenerative capacity of muscle cells, worsening myasthenic symptoms. As a result, therapeutic strategies aimed at improving mitochondrial function are emerging as a promising focus in MG research.

##### Changes in mitochondrial membrane dynamics in MG

Mitochondrial membrane dynamics involve fusion, fission, and the remodeling of cristae and other anatomical structures [26]. Mitochondrial fusion allows the exchange of contents between mitochondria, helping them resist damage from oxidative stress [27]. Mitochondrial fission facilitates the creation of new mitochondria and the removal of damaged or dysfunctional ones, supporting mitochondrial quality control. During extreme oxidative stress, fission also promotes apoptosis. These processes are constantly occurring within cells to maintain mitochondrial function, support respiration, and coordinate the tricarboxylic acid cycle, thus regulating cellular metabolism [28]. Additionally, mitochondrial dynamics play a role in regulating complex signaling events related to cell proliferation, differentiation, aging, and death.

**Figure 1.**
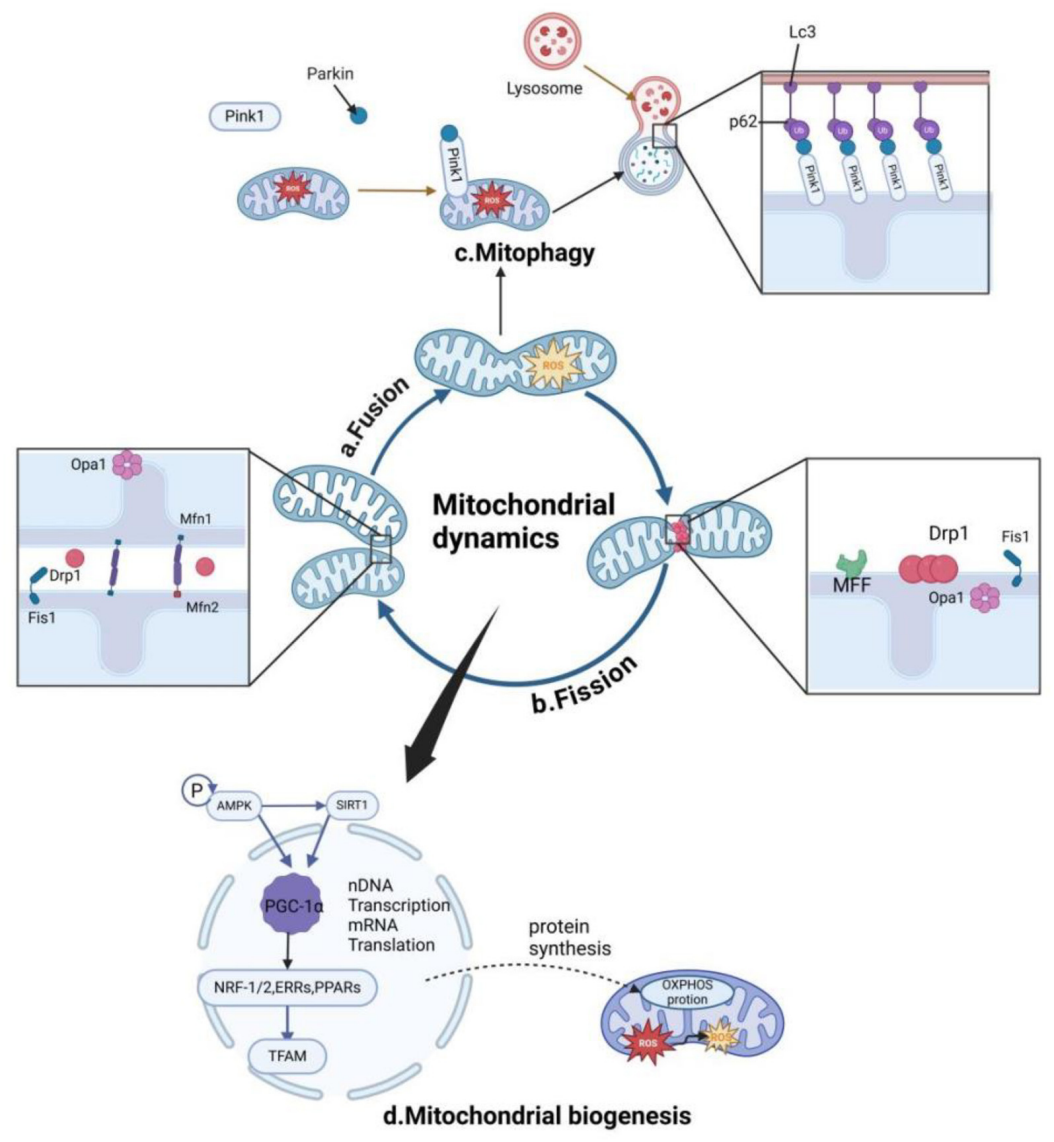
**Regulation of Mitochondrial Quality Through Mitobiogenesis and Mitophagy.** Mitochondria have a limited lifespan and must be constantly renewed to maintain normal function. Mitochondrial quality is tightly regulated by two fundamental, opposing mechanisms: mitobiogenesis and mitophagy. These processes respond to cellular energy demands as well as various cellular and environmental cues. The balance between mitochondrial fusion and fission is essential for both mitobiogenesis and mitophagy.

The processes of mitochondrial fusion and fission are tightly regulated by proteins, such as mitofusin 1 and 2, optic atrophy type 1 (Opa1), dynamin-related protein 1 (Drp1), and Fis 1 [29]. These proteins, located in both the outer and inner mitochondrial membranes, enable mitochondria to merge or divide as needed. Mitofusin 1 and 2, found in the outer mitochondrial membrane (Figure 1A and 1B), regulate mitochondrial fusion [30], while Opa1, located in the inner membrane near the cristae, is part of the dynamin protein family and plays a vital role in maintaining the structural integrity of the inner membrane. Opa1 also prevents proton leakage and helps facilitate electron transport between respiratory chain complexes. Together with mitofusin 1 and 2, Opa1 is critical for controlling cristae morphology [31] and synergistically regulates mitochondrial fusion [32].

Drp1, a protein that normally floats in the cytoplasm, translocates to the mitochondrial surface when it receives specific signals. There, it interacts with Fis 1, a protein anchored in the outer mitochondrial membrane, to initiate mitochondrial fission [32, 33]. Drp1 plays a bidirectional role in maintaining mitochondrial morphology. Increased Drp1 activity promotes mitochondrial division, while the expression of a dominant-negative mutant form of Drp1 (Drp1K38A) prevents mitochondrial fragmentation and reduces apoptosis [34]. Conversely, downregulation of Drp1 can lead to decreased mitochondrial membrane potential and reduced ATP production [35]. Research by Mouli et al. [36] found that during fusion events, mitochondrial components can be unevenly distributed, resulting in the parent mitochondrion producing two distinct daughter mitochondria through asymmetric fission.

Mitochondrial fusion and fission also affect energy metabolism, as changes in the expression of these regulatory proteins directly impact mitochondrial processes. Opa1 is crucial for maintaining the stability of mitochondrial cristae, and homozygous mutations in this gene can disturb cristae structure, leading to mitochondrial hypertrophy [37]. Th17 cells, a subset of autoreactive helper T cells involved in autoimmune inflammation [38], also show signs of mitochondrial abnormalities. Th17 cells producing IFN-γ, considered pathogenic, are thought to drive autoimmune inflammation by stimulating B cells to produce antibodies or by disrupting the balance of Th1/Th2 cytokines involved in neuromuscular transmission [6, 39]. A study found [28] that Th17 cells have mitochondria that are fused together with closely arranged cristae. Th17 cells lacking Opa1 rely on Opa1 to regulate the tricarboxylic acid cycle. Opa1 has emerged as a critical factor in the function of Th17 cells, while serine–threonine liver kinase B1 acts as a sensor that connects mitochondrial signals to Th17 cell activity.

In EAMG rats, the levels of mitofusin 1, mitofusin 2, Opa1, Drp1, and Fis 1 proteins in skeletal muscle tissues are significantly reduced compared to normal levels. This disruption of mitochondrial fusion and fission affects skeletal muscle cells [37] and compromises mitochondrial function. Membrane dynamics are fundamental to the overall physiological function of mitochondria, and the balance between fusion and fission provides the necessary environment for normal mitochondrial activity. Research in this area is crucial, and future studies should build on existing work to further explore the expression of fusion- and fission-related genes and proteins in MG patients. By regulating these imbalanced mitochondrial dynamics, it may be possible to alleviate or reverse the muscle damage caused by MG.

##### The significant impact of mitophagy in MG

When mitochondria become damaged, they divide unevenly and are identified by autophagosomes, which return to the cytoplasm to be broken down in lysosomes. This process, known as mitophagy, is essential for removing malfunctioning mitochondria and maintaining mitochondrial and cellular balance. Mitochondrial depolarization, regulated by factors, such as ROS, nutrient scarcity, and cellular aging, is a necessary condition for mitophagy. The fusion and fission receptor, Drp1, undergoes phosphorylation by 5’-AMP-activated protein kinase (AMPK), which is crucial for mitochondrial fission and the subsequent removal of damaged mitochondria via mitophagy [40]. Impaired mitochondria are sequestered in autophagic vesicles, which are then broken down by lysosomal enzymes, ensuring the functionality of mitochondria by recycling components [41].

Research has identified two main mechanisms of mitophagy: the ubiquitin-dependent and ubiquitin-independent pathways [42]. Figure 1C shows the well-known pathway in the ubiquitin-dependent mechanism, known as the PINK1 (phosphatase and tensin homolog-induced kinase 1)/E3 ubiquitin ligase (Parkin) system [43]. PINK1, a mitochondrial protein, is regularly imported into the inner membrane of healthy mitochondria and subsequently degraded [44], making it undetectable. However, when the mitochondrial membrane potential is disrupted, PINK1 can no longer enter the inner membrane and begins to accumulate on the outer mitochondrial membrane. Following mitochondrial damage, Parkin undergoes structural changes, exposing its catalytic cysteine and activating it as an E3 ubiquitin ligase. Normally located in the cytoplasm, Parkin is rapidly recruited to damaged mitochondria under mitochondrial stress. Phosphorylation of ubiquitin ligase p62 is also triggered, facilitating the interaction between p62 and LC3 in mitochondria, which triggers mitophagy [45].

Parkin plays a critical role in maintaining mitochondrial quality. It preserves mitochondrial network integrity by promoting the division of damaged mitochondria and degrading proteins involved in mitochondrial fusion, thereby preventing the fusion of impaired mitochondria [46]. Disruption of the Parkin gene [47] leads to the deterioration of skeletal muscle fibers and severe mitochondrial dysfunction. Overexpression of Parkin [48] can increase mitochondrial abundance in skeletal muscle, enhance mitochondrial enzyme activity, mitigate age-related muscle mass and strength decline, and reduce oxidative stress. Thus, Parkin is key to preserving mitochondrial function in skeletal muscle. Previous research has also shown that motor neuron degeneration and the NMJ are affected by mitophagy, making mitophagy essential for maintaining NMJ function [49, 50]. In the future, targeting mitophagy may be a potential therapeutic strategy for restoring NMJ function. Studies have shown that expression levels of mitophagy markers PINK1 and Parkin mRNA and protein are reduced in the skeletal muscles of EAMG rat models, while p62 expression is elevated [51].

Autophagy plays a role in all stages of T cell development and differentiation [52]. It protects activated T cells from oxidative damage by maintaining normal mitophagy, which rapidly eliminates excess or damaged mitochondria. Additionally, autophagy supports normal energy metabolism, which is vital for T cell homeostasis and optimal immune function [53]. Watanabe et al. [54] found that CD4^+^ T lymphocytes lacking autophagy are more susceptible to apoptosis via mitochondrial pathways. The failure of mitophagy leads to an increase in apoptosis, as abnormal mitochondria accumulate and increase ROS levels in T cells, causing cellular damage that can trigger disease. Activating mitophagy can remove defective mitochondria resulting from ROS generation [55, 56]. Studies on mice with *VPS34* or *Atg7* gene deletions [57] have shown decreased autophagy in CD4^+^ T cells, leading to impaired mitochondrial clearance and an excessive buildup of ROS.

Regulatory T (Treg) cells, which express CD4, CD25, and FOXP3, are a specialized subset of T cells that regulate the immune system. Tregs inhibit the activation of autoreactive T and B cells. In MG, Treg cells show imbalances in their number and/or function [58]. Impaired mitophagy in Tregs leads to the accumulation of damaged mitochondria, altered energy metabolism, and an abnormal intracellular environment, all of which hinder Treg activation and proliferation, leading to a loss of self-tolerance. These factors contribute to the onset and progression of MG. Multiple studies have demonstrated that disturbances in Treg equilibrium and functionality are key contributors to MG development [59]. Prior research [60, 61] has also shown that Treg suppression is diminished in MG patients, and mitophagy plays a significant role in T cell fate. Mitophagy is intricately linked to the maturation, activation, and specialization of T cells, making it crucial to monitor mitophagy in Tregs from MG patients.

**Figure 2.**
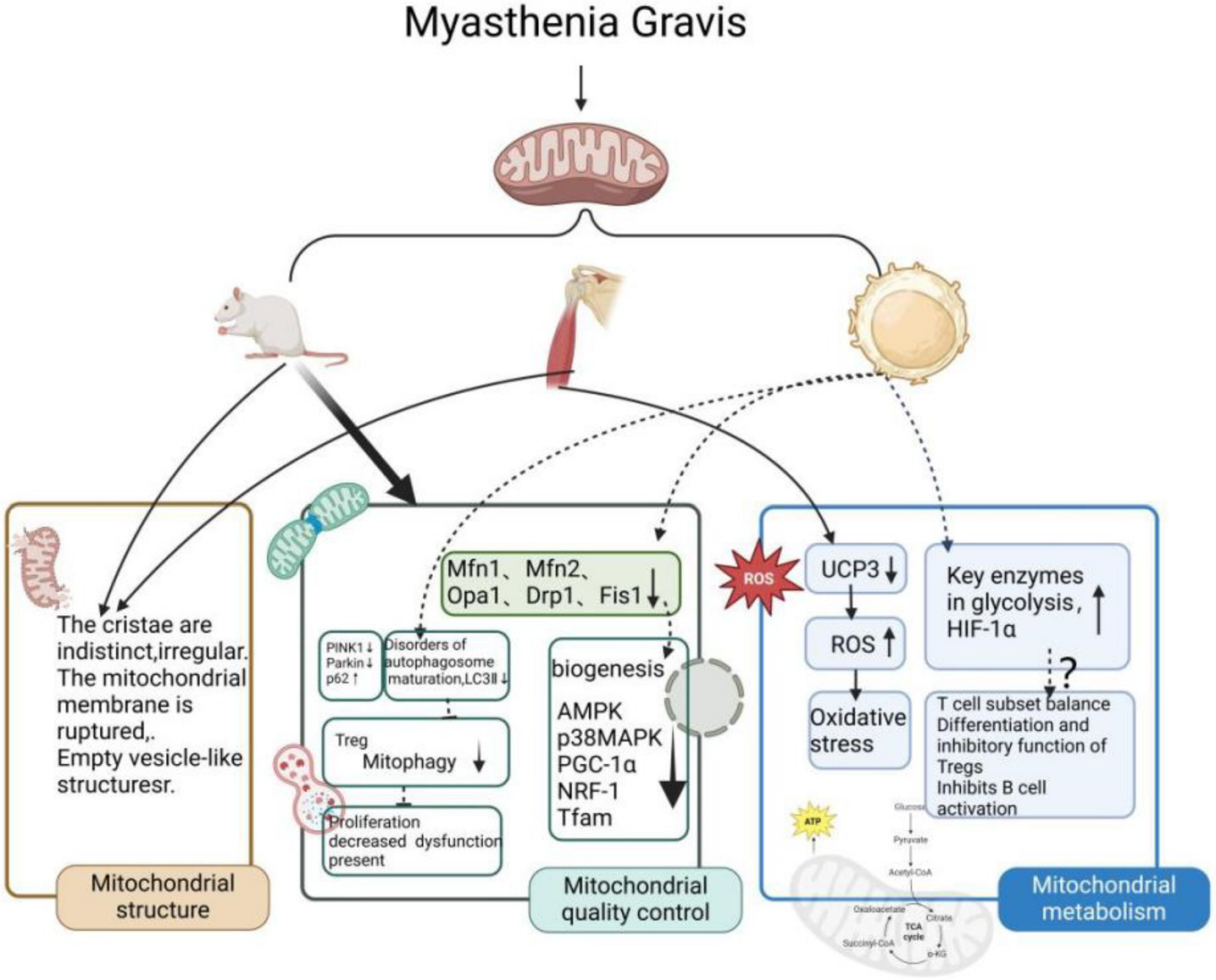
**Pathological changes of mitochondria in MG and possible pathogenic mechanisms**. To date, research on the role of mitochondria in the pathology of MG has primarily focused on EAMG rat muscles, MG patient muscles, and immune cells. Mitochondrial alterations are a consequence of MG and involve structural changes. Mitochondria contribute to the onset and progression of MG, with mitochondrial quality control and metabolism playing critical roles. Further investigation into mitochondria could eventually uncover the upstream mechanisms behind MG pathogenesis. MG: Myasthenia gravis; EAMG: Experimental autoimmune myasthenia gravis.

As shown in Figure 2, MG patients exhibit impaired mitophagy in Tregs, resulting in elevated ROS production, accumulation of damaged mitochondria, disrupted energy metabolism, and an abnormal intracellular environment [62]. The decline in mitochondrial membrane potential and mitophagy in Treg cells may be associated with decreased proliferation and diminished suppressive capacity. Research by Wang et al. [63] found that Tregs in MG patients show reduced mitophagosome formation, decreased mitophagy, and lower levels of the autophagy-related protein LC3II. In MG patients, the mitophagy levels in peripheral blood Tregs are reduced, and abnormal mitochondria are not effectively cleared by mitophagy. This results in increased levels of ROS and toxic mitochondrial components, which ultimately impair Treg function and hinder Treg cell growth.

Mitophagy plays a crucial role in improving mitochondrial dysfunction, which is necessary for maintaining metabolic homeostasis and clearing damaged mitochondria. It is also vital for cell differentiation and proliferation. While most mitophagy research has focused on Treg cells, MG is characterized by imbalances in various T lymphocyte subsets. Future studies should investigate the state of mitophagy in different cell types in MG to better understand its role in the disease and explore potential therapeutic strategies.

##### Alterations in mitochondrial biogenesis in MG

Mitochondrial biogenesis is crucial for cellular energy production, involving the continuous regulation, degradation, and synthesis of organic molecules. It is controlled by various factors, such as cellular energy levels, growth hormones, metabolites, and oxidative stress. Processes like mitochondrial fission, fusion, and mitophagy support mitochondrial biogenesis, which requires the coordinated expression of both mitochondrial and nuclear genomes. This coordination is critical because mitochondria possess their own genome, known as mitochondrial DNA (mtDNA).

Mitochondrial biogenesis is tightly regulated by upstream energy metabolism pathways, such as SIRT1 and AMPK, as illustrated in Figure 1D. SIRT1, part of the NAD^+^-dependent histone deacetylase family, acts as a sensor for cellular energy status [64]. It is positively regulated by oxidized NAD^+^ and influences the expression of peroxisome proliferator-activated receptor gamma coactivator 1-alpha (PGC-1α) and other mitochondrial-related genes, making it a significant focus in mitochondrial research [65]. AMPK, a master regulator of cellular energy metabolism, is activated when the AMP/ATP ratio increases [66]. Physical activity can stimulate AMPK, which in turn promotes mitochondrial biogenesis by phosphorylating PGC-1α. AMPK also enhances its own deacetylation through SIRT1, creating a feedback loop that boosts its expression [67]. This interaction underscores the importance of the AMPK/PGC-1α axis in maintaining energy balance and regulating mitochondrial metabolism [68].

The downstream players involved in mitochondrial biogenesis include nuclear respiratory factors (NRFs), peroxisome proliferator-activated receptor gamma (PPARγ), and PGC-1α. NRF-1 regulates the transcription of nuclear genes essential for mitochondrial respiration. These transcription factors promote the expression of genes encoding subunits of the mitochondrial respiratory chain complexes. PGC-1α interacts with transcription factors like NRF1 and NRF2 to regulate the expression of mitochondrial genes and the synthesis of proteins needed for mtDNA transcription, such as transcription factor A (TFAM). These interactions ensure proper mitochondrial metabolism and biogenesis by regulating mtDNA transcription [68, 69].

SIRT1, AMPK, and PPARγ regulate mitochondrial biogenesis through PGC-1α, increasingly recognized as a central hub for energy metabolism. PGC-1α links the creation of new mitochondria with processes like mitochondrial fusion and fission, and it can reduce mitochondrial oxidative stress. AMPK can regulate PGC-1α activity indirectly, including through the activation of P38 MAPK, which depends on AMPK [70]. P38 MAPK is critical for AMPK-induced PGC-1α expression during metabolic stress, with both AMPK and P38 MAPK playing roles in regulating PGC-1α levels. Chronic nutrient overconsumption can disturb cellular energy homeostasis, leading to reduced AMPK expression and impaired PGC-1α activity, which in turn contributes to mitochondrial dysfunction [71, 72]. Therefore, PGC-1α, along with its upstream regulators AMPK and P38 MAPK, are key therapeutic targets for improving clinical outcomes in MG.

As shown in Figure 2, Ke et al. [73] proposed a link between mitochondrial biogenesis signaling pathways and MG, finding that mitochondrial-related protein levels were lower in the peripheral blood of MG patients, suggesting that changes in mitochondrial dynamics and biogenesis are associated with the disease. This supports the idea that mitochondrial markers could be useful for diagnosing MG, especially in patients who test negative for anti-AChR or anti-MuSK antibodies [69, 70]. Skeletal muscle, which makes up about 40% of body weight, is highly metabolically active and plays a vital role in energy balance and physical movement. In EAMG rat models, reduced levels of p38 MAPK, AMPK, PGC-1α, NRF-1, and TFAM have been observed in the gastrocnemius muscle [74, 75]. This suggests that mitochondrial gene expression and protein synthesis are impaired in both MG and EAMG, leading to reduced mitochondrial abundance and energy metabolism—critical for normal muscle function. Mitochondrial biogenesis, driven by energy metabolism, is vital for maintaining mitochondrial function and overall energy balance, highlighting its significant role in MG pathophysiology.

##### The crucial role of mitochondrial metabolism in MG

In eukaryotic cells, energy conversion is essential for generating cellular biomass and maintaining cellular functions. Energy metabolism involves continuous cycles where ATP supplies the energy required by cells. Mitochondria are central to this process, producing ATP through OXPHOS in the inner mitochondrial membrane. During cellular respiration, various substrates are oxidized, resulting in electron transfer to molecular oxygen, which is reduced to water. A sequence of enzyme complexes supports this electron transport chain. The cristae of the inner mitochondrial membrane house these complexes, making them key sites for OXPHOS and mitochondrial respiration [16].

Mitochondria are the primary source of cellular energy, particularly in highly metabolically active tissues like muscle, which require efficient energy production for contraction and other activities. Mitochondrial dysfunction can disrupt normal energy metabolism and exacerbate neuromuscular disorders [76]. Kordas et al. [77] highlighted the importance of the mitochondrial protein CHCHD10 in ATP production, noting that it enhances AChR expression and supports agrin-induced AChR clustering.

##### Oxidative stress in MG muscles

Oxidative stress occurs when ROS production exceeds the cell’s ability to neutralize and eliminate them, leading to an imbalance between oxidation and reduction processes. Under normal conditions, cells maintain a dynamic equilibrium between pro-oxidants and antioxidants, with concentrations fluctuating depending on factors, such as cell type, mitochondrial activity, redox-related protein expression, and external factors [78, 79]. In pathological conditions, an imbalance in redox reactions and elevated ROS levels can lead to intracellular oxidative stress, damaging DNA, proteins, and lipids in the plasma membrane [80]. Since mitochondria generate most of the cellular ROS, they are a key source of oxidative stress [81].

In MG, mitochondrial ROS production has primarily been studied in skeletal and EOMs. Skeletal muscle is essential for movement and maintaining metabolic balance [82]. During respiration and OXPHOS, mitochondria use oxygen to generate ATP and ROS. Excessive ROS production can lead to oxidative stress, damaging mitochondrial respiratory chain enzyme complexes, particularly those on the inner mitochondrial membrane [83]. In MG, the loss of mitochondrial respiratory chain complex I is [73], common, underscoring the importance of an effective antioxidant defense system for cellular survival under aerobic conditions [84].

As shown in Figure 2, muscle pathology in MG reveals that muscles, other than EOMs, often show neurogenic changes regardless of the MG serotype. Mitochondrial stress or damage is prevalent in MG muscles. Notably, Cenacchi et al. [21] observed more prominent neurogenic alterations in patients with AChR-MG, as evidenced by mitochondrial stress in all samples. EOMs have higher concentrations of mitochondria compared to other muscles, highlighting the critical role of mitochondrial function in these muscles, which require continuous energy for their high firing rates. Research has shown that mitochondrial uncoupling protein 3 expression is significantly reduced in OP-MG muscle cells compared to control MG cells. Uncoupling protein 3 may have a protective role in conditions like EAMG by reducing ROS formation and preserving mitochondrial function [85]. The lower levels of this protein in OP-MG muscle cells could weaken the defense against oxidative stress.

Besides the role of MuSK and AChR antibodies, voltage-gated potassium (KV1.3) channels also significantly impact MG. KV1.3 is a transmembrane protein that forms channels allowing potassium ions (K^+^) to move across the plasma membrane, maintaining cellular homeostasis. This function may be particularly important in MG [86].

The exact cause of the OP-MG subtype remains unclear. Mitochondrial function is especially vital in EOMs due to their high energy demands. Histopathological studies show that both EOMs and skeletal muscles in MG patients experience mitochondrial stress. However, it is uncertain whether this stress is a fundamental cause of the disease or a consequence of muscle weakness [87]. Genetic association studies and gene profiling of muscle cells from MG patients suggest that mitochondrial metabolic pathways contribute to the development of OP-MG [88].

Glucose serves as the primary fuel for immune cells, and its metabolic processing varies among different immune cell types and between their inactive and active phases [89]. Glucose metabolism can proceed through two main pathways: OXPHOS and anaerobic glycolysis. Glycolysis typically produces a net of two ATP molecules, whereas OXPHOS generates 36 ATP molecules per mole of glucose. OXPHOS refers to the process by which ATP is produced through biological oxidation, primarily during electron transport in the respiratory chain. Ninety-five percent of ATP in living organisms is generated this way. Glycolysis, on the other hand, occurs in the cytoplasm under anaerobic conditions, breaking down glucose into pyruvate and producing two ATP molecules per glucose molecule. It involves three key rate-limiting enzymes: hexokinase, phosphofructokinase, and pyruvate kinase. The PI3K/Akt, JAK/STAT, and mTOR signaling pathways promote metabolic switching in immune cells, with the PI3K/Akt pathway activating mTOR, which upregulates glycolytic enzyme activity and increases glucose transporter expression on the cell membrane [90, 91].

Several studies emphasize the vital role of glucose metabolism in the differentiation and function of immune cells, such as conventional T cells, Tregs, DCs, and B cells. The availability and usage of metabolic substrates are essential for effectively regulating the activity of these cells [92, 93]. For example, immature DCs primarily rely on mitochondrial OXPHOS. Upon activation, they undergo metabolic reprogramming, shifting to glycolysis, which becomes the primary ATP source necessary for rapid cell growth and the production of critical immune-modulating molecules. This metabolic shift, where glucose is converted to lactate even in the presence of oxygen, is known as the Warburg effect or aerobic glycolysis [94, 95].

Resting naive CD4^+^ T cells predominantly rely on mitochondrial lipid oxidation, glucose oxidation, and fatty acid oxidation via the OXPHOS pathway to meet their energy needs. Upon activation, T cells upregulate hypoxia-inducible factor 1-alpha (HIF-1α), even under normoxic conditions, mimicking metabolic changes seen in DCs. Th1 and Th17 cells also shift toward aerobic glycolysis upon activation [96, 97]. Meanwhile, naive B cells, upon activation by helper T cells, exhibit a significant increase in both glycolysis and OXPHOS, which is essential for plasma cell development and antibody production [98].

T-cell dysfunction or apoptosis can result from mitochondrial impairment, which involves decreased mitochondrial clearance and increased ROS production. The metabolic state of T cells is intricately linked to the number and condition of their mitochondria [99]. Studies on mice with *VPS34* or *Atg7* gene deletions have shown that a decrease in autophagy in CD4^+^ T cells leads to impaired mitochondrial clearance and increased ROS generation, highlighting the critical role of these processes in preserving T-cell function. Treg cell development, in particular, depends on mitochondrial lipid oxidation, whereas effector T cells rely on glycolysis. OXPHOS remains the primary energy source in specialized immune cells [29]. In Tregs, mitochondrial metabolism is vital for supporting their immunosuppressive function [100] and stability [101], allowing them to survive in lactate-rich environments [102].

As an autoimmune disease, the pathogenesis of MG involves antigen-presenting cells recognizing antigens and activating autoreactive T cell subsets. Metabolic reprogramming is necessary for CD4^+^ T cell differentiation to meet the energy demands of cell division and effector function. Disruptions in T-cell subset distribution trigger the activation of autoreactive B cells and the production of pathogenic antibodies. This process is characterized by complex immunometabolic changes, where metabolic abnormalities initiate a cascade of pathogenic processes, leading to the symptoms of muscle weakness seen in MG.

As shown in Figure 2, the glycolysis pathway plays a critical role in regulating immune cell alterations in MG. Research by Li et al. [103] discovered unique patterns of glucose metabolism among different immune cell subtypes in MG. Antigen-presenting cells, Th1, Th17, and B cells in MG patients show increased glycolysis and elevated expression of key glycolytic enzymes, including HIF-1α. This suggests that the mTOR-HIF-1α signaling pathway may serve as a regulatory node for immunometabolic reprogramming in MG. Glycolysis and its regulatory pathways influence the immune functions of DCs, contribute to the balance of CD4^+^ T cell subsets, regulate the development and function of Tregs, and modulate B cell activation. Following activation through B cell receptors or lipopolysaccharides, both glycolysis and OXPHOS provide the energy needed by activated B cells. This study highlights the importance of metabolic pathways, including glycolysis, in the immune response of MG patients and identifies potential therapeutic targets.

Mitochondria play a crucial role in the function of Treg cells and the pathogenesis of MG. However, the exact mechanisms through which mitochondria affect Treg alterations and MG development remain incompletely understood. Further research is needed to elucidate how mitochondrial activity influences histone modifications and attracts transcription factors to the Foxp3 promoter and regulatory regions. Moreover, research has shown that glucose metabolism is essential for stimulating the development of human T and B cells [104] and regulating their migration [105]. It is important to investigate whether altering metabolic conditions can impact the activation, differentiation, and migration of immune cells in autoimmune diseases like MG.

The underlying mechanisms behind mitochondrial dysfunction in the immune cells of MG patients are still unclear, but they may play a crucial role in disease regulation. More research is needed on the immunometabolic reprogramming of antigen-specific B cells and antibody production, as B cell activation involves complex metabolic patterns and regulatory mechanisms intertwined with other immune cells. Another key area for investigation is how external factors, such as medications, diet, and environmental influences, regulate mitochondrial metabolism in immune cells.

### Research findings on mitochondria-related drugs

The non-selective immunosuppression by oral steroids and non-steroidal immunosuppressants remains a central component of MG treatment. Although therapies aimed at improving NMJ, transmission and inhibiting autoimmune responses have shown some efficacy, they come with drawbacks, particularly in failing to improve or even exacerbating muscle cell damage. Over the past decade, mitochondria have gained increasing attention as a potential therapeutic target.

#### The effects of current MG treatments on mitochondria

##### Effects of common immunosuppressive drugs on mitochondria

The most commonly used immunosuppressants in MG include azathioprine, mycophenolate mofetil, methotrexate, cyclosporine, and tacrolimus. Many centers employ cyclophosphamide and rituximab for refractory MG or cases associated with muscle-specific receptor tyrosine kinase antibodies. However, these immunosuppressants can negatively affect mitochondrial function, potentially impacting T-cell differentiation and function by reducing energy production, increasing toxic ROS generation, and inducing apoptosis.

Glucocorticoids [106] play a complex role in regulating mitochondrial function. For instance, research by Stefania showed that short-term administration of glucocorticoids in Parus major nestlings activates protective mechanisms, positively correlating with mitochondrial biogenesis and enzyme activity. However, long-term use can lead to mitochondrial dysfunction, characterized by impaired respiratory chain function, reduced ATP production, increased ROS generation, structural abnormalities, Drp1 phosphorylation-induced fission, impaired calcium buffering, and excessive mitochondrial ROS production. These changes increase cellular sensitivity to death and telomere attrition [107]. Additionally, glucocorticoids can directly affect the mitochondrial genome.

In MG patients, carbonyl cyanide m-chlorophenyl hydrazone (CCCP) promotes mitophagy in Tregs, clearing damaged mitochondria, reducing metabolic stress, and decreasing apoptosis. Conversely, the mitophagy inhibitor cyclosporine A exacerbates metabolic disorders and increases apoptosis by inhibiting mitophagy in Treg cells of MG patients [62]. Research has also shown that cyclosporine A reduces the expression of PGC-1α mRNA and protein levels in HepG2 cells, thereby inhibiting mitochondrial biogenesis [108]. Similarly, in mice exposed to cyclophosphamide, mitochondrial dysfunction and oxidative stress may lead to muscle damage [109].

Metformin, another drug under investigation, demonstrates anti-inflammatory properties through the activation of AMPK. Studies conducted on EAMG rat models have demonstrated that oral administration of metformin can mitigate the severity of the condition by rectifying the dysregulation of several T cell populations [110].

In addition to classic immunosuppressants, targeted immunotherapies [111] aim to treat MG by blocking the complement system or neonatal Fc receptor, thereby preventing the circulation of immunoglobulins and reducing immunoglobulin G antibodies, such as with efgartigimod. These treatments can successfully reduce the mechanisms of the disease temporarily; however, they do not permanently cure autoimmune diseases. Additionally, inhibiting the complement cascade increases the risk of other infections. Further research is needed to fully understand the long-term effects of these drugs on immune cells in the context of MG. Cellular metabolism [112], closely linked to mitochondrial function, plays a key role in determining the fate and function of immune cells.

##### Effects of new drugs on mitochondria

Recent research has discovered that different medications can enhance mitochondrial function in individuals with MG. Astragaloside IV (AS-IV), a naturally occurring substance extracted from *Astragalus*, has been found to effectively decrease blood AChR-Ab levels in rats with EAMG. Additionally, AS-IV has been shown to significantly improve the structure and function of skeletal muscle mitochondria [51]. It enhances mitochondrial function by inhibiting the decline of mitochondrial membrane potential and ATP while also elevating nitric oxide and PGC-1α levels, which are crucial for stimulating mitochondrial biogenesis [113].

Recent studies have confirmed that AS-IV promotes mitochondrial autophagy via the PINK1/Parkin pathway, enhances the efficacy of superoxide dismutase in eliminating superoxide, and reduces cellular oxidative stress. Furthermore, AS-IV mitigates mitochondrial damage by eliminating excessive ROS, enhances ATP production capacity, and helps maintain normal mitochondrial activity [114].

Zhang et al. [115] utilized molecular docking technology to investigate the potential therapeutic mechanisms of the Qishen Dihuang formula in managing MG. Their research indicates that the active components—quercetin, epigallocatechin-3-gallate, luteolin, kaempferol, and fisetin—may regulate the PI3K/AKT signaling pathway. These components could mitigate the progression of MG by inhibiting glycolysis via the PI3K/AKT/mTOR/HIF1α signaling pathway. Experimental validation using EAMG rats [116] has confirmed these findings.

Further research by Song [23] demonstrated that the therapeutic effects of Qiáng Jı- Jiàn Lì Tāng are mediated through the activation of the AMPK/PGC-1α signaling pathway, which enhances mitochondrial biogenesis and reverses decreased expression levels of related transcription factors in the gastrocnemius muscle of EAMG rats. This formulation can also alleviate pathological damage, improve muscle atrophy in MG, restore muscle energy supply, and enhance the contractile force of the gastrocnemius muscle. The research team suggested that Qiáng Jı- Jiàn Lì Tāng alleviates MG by improving energy metabolism [1]. This is achieved through regulating mitochondrial energy fusion and fission, potentially by enhancing the enzymatic activity of respiratory chain complexes. Qiáng Jı- Jiàn Lì Tāng also increases the expression of mitofusion 1 and 2, Opa1, Drp1, and fission 1 mRNA and proteins in EAMG rats, enhancing the frequency of fusion and fission events. This accelerates the isolation and removal of damaged mitochondrial materials, preserving mitochondrial morphology and biological function.

Additionally, research by Hu et al. based on the “spleen-mitochondria” theory has shown significant improvements in the ultrastructure of skeletal muscle, mitochondria, and NMJs in EAMG model rats after treatment with spleen-strengthening and qi-enhancing methods. These treatments also led to the marked alleviation of muscle weakness symptoms [117].

##### Other research findings on drugs aimed at improving mitochondrial function

Current research shows that various drug components can improve mitochondrial function, which may inspire future mitochondrial therapies for MG.

##### Achievements of drug monotherapies that improve mitochondrial function

Hesperidin [118] has demonstrated the ability to improve mitochondrial enzyme activity, reduce mitochondrial dysfunction, and strengthen antioxidant defense systems in APPswe/PS1dE9 transgenic mice, a model for Alzheimer’s disease. Similarly, ferulic acid has shown the capacity to enhance mitochondrial biogenesis and dynamics in a mouse model of vascular damage and to stimulate muscle growth [119]. Song et al. [23] discovered that atractylenolide III boosts the production of PGC-1α and markers of mitochondrial biogenesis, such as NRF-1 and Tfam, thereby increasing ATP content and improving energy metabolism in C2C12 myotubes.

Saikosaponin A [120], a triterpenoid saponin from *Bupleurum*, has exhibited anti-inflammatory, antioxidant, and cardioprotective effects in mouse models of cardiac remodeling and fibrosis, as well as in rat cardiomyocyte fibrosis models. It helps to reduce cardiac dysfunction and repair myocardial tissue damaged by prolonged stress. Liquiritin has shown neuroprotective effects, increased cellular antioxidant capacity, reduced mitochondrial damage, and improved mitochondrial quality control in mice with myocardial fibrosis [121, 122]. Zhao et al. [123] found that ammonium glycyrrhizinate can restore mitochondrial damage, reduce oxidative stress, and decrease ROS production, as measured by superoxide dismutase and GSH activities, MDA concentration, histological inspection of cardiac tissue, and changes in mitochondrial ultrastructure.

Mitoquinone [124], a widely used mitochondria-targeted antioxidant, has improved muscle atrophy, weakness, and oxidative metabolism in C26 tumor-bearing mice, enhancing muscle function and promoting balanced energy metabolism in skeletal muscles.

##### New approaches based on immunometabolism

Research in immunometabolism has shown that ROS from mitochondria play a significant role as signaling molecules, affecting the function and fate of T cells. Targeting glycolysis or ROS production to specifically eliminate autoreactive T cells while preserving overall immune function is a promising approach for treating autoimmune diseases. Enhancing the activity of immunosuppressive Treg cells could help improve self-tolerance. For example, butyrate, a byproduct of gut microbiota, influences Treg cell energy metabolism by impacting both OXPHOS and glycolytic pathways, thus regulating T cell differentiation. In AChR-positive MG patients, butyrate has been shown to restore damaged Treg cells through mTOR-mediated autophagy [125].

Suppressing the effector responses of cytotoxic CD8^+^ T cells can be achieved by reducing glycolytic activity or enhancing antioxidant defenses with molecules like glutathione and superoxide dismutase [126]. Additionally, Wang et al. [63] proposed that targeting Treg cell dysfunction with MitoTEMPO, a specialized mitochondrial superoxide scavenger, could reduce mitochondrial ROS production, minimize DNA damage, induce Treg apoptosis, and restore lysosomal function. These findings highlight the potential of targeting mitochondrial ROS and immunometabolic reprogramming as therapeutic strategies for T cell-mediated autoimmune diseases.

## Conclusion

Recent studies increasingly emphasize the crucial role mitochondria play in the development and progression of MG. Mitochondrial functions are complex, contributing to cellular bioenergetics, redox regulation, and intracellular signaling. Abnormalities in mitochondrial dynamics, biogenesis, and mitophagy have been observed in the peripheral blood of MG patients, pointing to significant mitochondrial quality control issues. While the precise mechanisms linking MG and mitochondrial dysfunction remain unclear, abnormalities in mitochondrial ultrastructure have been confirmed in the orbital and skeletal muscles, particularly in the EOMs of MG patients.

Future drug development should focus on restoring mitochondrial function to alleviate muscle fatigue. Mitochondria-targeted therapies may be particularly beneficial for MuSK antibody-positive MG patients, who show more pronounced mitochondrial abnormalities leading to muscle atrophy. 

The immune dysregulation in MG primarily involves dysfunctional T cells, autoreactive B cells, and the production of autoantibodies affecting multiple effector cells. The proliferation, differentiation, and activities of T and B cells rely on various mitochondrial metabolic pathways, though the specifics of these metabolic processes remain poorly understood. In particular, mitochondrial lipid oxidation in immune cell differentiation requires further investigation. Dynamic shifts in mitochondrial metabolism across immune cells are likely important but are currently only observed at isolated moments, underscoring the need for long-term studies.

Mitochondrial quality control and metabolic dysregulation in T lymphocyte subsets play a pivotal role in MG onset and progression. Many studies on autoimmune diseases highlight the importance of mitochondrial function in immune cells. Future research could focus on targeting mitochondrial processes to regulate T lymphocyte differentiation, proliferation, and normal physiological functions, which could provide new therapeutic avenues for treating MG.

Mitochondria contain multiple copies of mtDNA, essential for producing ribosomal and transfer RNAs, along with key proteins involved in OXPHOS. Dynamic processes like mitochondrial fusion and fission govern the distribution and maintenance of mtDNA. While research exists on transcription factors that promote mtDNA expression, the extent of mtDNA damage in MG and its role in the disease remain unclear. Investigating this could be a key focus for future MG research.

## Data Availability

Data sharing is not applicable to this article as no datasets were generated or analyzed during the current study.
